# A fast, efficient and high-throughput procedure involving laser microdissection and RT droplet digital PCR for tissue-specific expression profiling of rice roots

**DOI:** 10.1186/s12860-020-00312-y

**Published:** 2020-12-10

**Authors:** Thibault Mounier, Sergi Navarro-Sanz, Charlotte Bureau, Lefeuvre Antoine, Fabrice Varoquaux, Franz Durandet, Christophe Périn

**Affiliations:** 1 CIRAD, UMR-AGAP, Université de Montpellier, Avenue Agropolis, F-34398 Montpellier Cedex 5, France; 2 IAGE Company, Avenue Agropolis, F-34398 Montpellier Cedex 5, France

**Keywords:** Rice, Root meristem, Laser microdissection (LM), Cortex, ddRT-PCR, Droplet digital PCR

## Abstract

**Background:**

In rice, the cortex and outer tissues play a key role in submergence tolerance. The cortex differentiates into aerenchyma, which are air-containing cavities that allow the flow of oxygen from shoots to roots, whereas exodermis suberification and sclerenchyma lignification limit oxygen loss from the mature parts of roots by forming a barrier to root oxygen loss (ROL). The genes and their networks involved in the cellular identity and differentiation of these tissues remain poorly understood. Identification and characterization of key regulators of aerenchyma and ROL barrier formation require determination of the specific expression profiles of these tissues.

**Results:**

We optimized an approach combining laser microdissection (LM) and droplet digital RT-PCR (ddRT-PCR) for high-throughput identification of tissue-specific expression profiles. The developed protocol enables rapid (within 3 days) extraction of high-quality RNA from root tissues with a low contamination rate. We also demonstrated the possibility of extracting RNAs from paraffin blocks stored at 4 °C without any loss of quality. We included a detailed troubleshooting guide that should allow future users to adapt the proposed protocol to other tissues and/or species. We demonstrated that our protocol, which combines LM with ddRT-PCR, can be used as a complementary tool to in situ hybridization for tissue-specific characterization of gene expression even with a low RNA concentration input. We illustrated the efficiency of the proposed approach by validating three of four potential tissue-specific candidate genes detailed in the RiceXpro database.

**Conclusion:**

The detailed protocol and the critical steps required to optimize its use for other species will democratize tissue-specific transcriptome approaches combining LM with ddRT-PCR for analyses of plants.

## Background

Aerenchyma formation is tightly developmentally controlled in rice roots. Moreover, aerenchyma are fragile tissues embedded between vascular and outer cell layers; thus, identifying gene networks involved in aerenchyma is challenging and requires the development of specific RNA extraction procedures for downstream applications such as RNAseq. Three main technologies have been developed for the isolation of RNA from specific tissues, and these can be grouped into two groups: technology involving laser microdissection (LM) [1] and FACS [2] and INTACT [3, 4] technologies.

The FACS and INTACT technologies use transgenic lines and specific tissue promoters that express fluorophores or a nuclei-tagged protein [2–4], respectively. Seedlings are then digested to produce protoplasts for FACS and nuclei for INTACT. These protoplasts and nuclei are separated into GFP-labeled and unlabeled populations, using a cell sorter (FACS) or an affinity column (INTACT). RNAseq or microarray is then used to obtain an expression profile of the labeled cell populations. These technologies have mainly been used for the analysis of *A. thaliana* (e.g., [2] and to a lesser extent for the analysis of rice (e.g., [5, 6]. For *A. thaliana*, the development of FACS technology has made it possible to produce a map of the expression profiles of most root cell types [2] and to analyze tissue-specific responses to salt stress [7].

The INTACT and FACS approaches require transgenic plants and tissue or cell-specific promoters. Moreover, for FACS, the protoplast isolation step generates biases and often cross-contamination that are sometimes difficult to control, and thus, a set of stress control experiments are needed [7]. In contrast, INTACT requires only frozen tissues for the isolation of nuclei through affinity purification [5]. These technologies cannot be used to isolate few cells from a specific tissue unless a specific promoter is available, require large quantities of biological material proportional to the number of labeled cells and are well suited for large-scale transcriptomics experiments.

LM technology is complementary to FACS and INTACT technologies. It involves the laser cutting of paraffin-embedded or frozen tissue sections for the extraction of specific RNAs that can be used to determine expression profiles using DNA chips or RNAseq (e.g., [8, 9]. This technology has been used for the analysis of a larger number of species because it does not require tissue-specific promoters or the generation of transgenic plants. Theoretically, it can be used for gene-specific expression profiling in small-scale experiments. In particular, this technology has been used to isolate root tissues from rice [1, 10]; however, the technology requires optimization of many parameters, such as those associated with fixation, dehydration, paraffin embedding, and laser steps [9], and has therefore been mastered only by a few laboratories.

In our first experiments using an LM-based approach to isolate RNA from the cortex of rice roots, we used available published protocols [8, 9, 11] but found that isolating good-quality RNA (RIN > 7) while maintaining an intact tissue structure was difficult. This finding encouraged us to re-perform each step to determine the key parameters and to rationally optimize each step by attempting to identify the main sources of variation in the quality and quantity of RNA and the tissue structure.

RNAs extracted through LM can be used to perform transcriptomic analyses by RNAseq or microarray or directly to determine the tissue-specific expression profiles of candidate genes. These expression analyses are most often performed by qPCR or RT-PCR but have several difficulties. The quantities of extracted RNA are extremely small, with results in the need for a large amount of tissues and/or the use of amplification systems that potentially introduce bias. qPCR is sensitive to potential contaminants and PCR inhibitors, and its reliability requires almost-perfect PCRs. Unfortunately, the most interesting samples are those containing small quantities of the targets, which can result in small or very small expression differences, and these samples are potentially contaminated by PCR contaminants present in paraffin samples [12].

Similar to qPCR, droplet digital PCR (ddPCR), which is a recently developed technology, uses Taq polymerase in PCRs to amplify the targets but has two important advantages compared with qPCR [13]. The PCRs are distributed in 20,000 independent droplets, and expression data are collected at the end of the PCR. These two differences allow direct quantification without a standard curve to obtain more accurate and repeatable results. The fluorescence measurements at the end of the reaction in each droplet (yes/no, hence the term digital PCR) enable expression quantification independent of the PCR efficiency [13]. RT-ddPCR can therefore also be used to measure the expression level of genes in samples containing extremely small quantities of the targets as well as PCR contaminants [12]. To the best of our knowledge, RT-ddPCR, despite its potential, has not yet been used to test gene expression in combination with LM.

We developed a simplified, high-throughput protocol involving the use of LM and ddPCR to extract high-quality RNA, control intertissue contamination, and analyze gene expression. We identified key steps and simple solutions for any research group wishing to use this protocol for other tissues of other species. We also obtained evidence showing that this protocol can be applied to samples with a low level of intertissue contamination through the use of tissue-specific markers. We demonstrated the possibility of storing paraffin samples without any loss of quality for at least 6 months, which would enable sample collection from plant species in the field. Finally, we showed that ddRT-PCR can be used to evaluate the tissue specificity of candidate genes directly from RNA extracted by LM, indicating that the proposed protocol can be considered a powerful and complementary tool to in situ hybridization and in situ RT-PCR. ddRT-PCR can also be used as quality control test before any downstream application such as RNAseq. Lastly, we illustrated the efficiency and novelty of our approach in determining tissue-specific expression using candidates extracted from the RiceXpro database [14, 15].

The complete protocol and the associated troubleshooting guide should make it possible to democratize approaches combining LM with ddRT-PCR for use in numerous applications associated with plant development.

## Results

### Summary of the LM protocol

The full protocol, including the critical steps (**notes**) and advice for researchers wishing to apply the protocol to other tissues or species, is detailed in Supplemental File 1 (see also Methods). Briefly, the first step constitutes germination of the rice seeds in an ARALAB (Supplemental Figure S1) using a hydroponics net floating system (Figure S1A), the subsequent collection of 2-cm root tips from 7-day-old seedlings and their overnight impregnation with fixative. Bundles of eight aligned roots are collected, and one root is stained with eosin (Figure S1B) to visualize the bundles in future paraffin blocks. In the second step, the root bundles are positioned in biopsy cassettes and trapped with biopsy foam (Figure S1C). After dehydration, the cassettes are immersed in a microwave water bath for embedding in paraffin. Finally, the bundles are positioned in liquid paraffin on a cold block (Figure S1D) and then soaked in the solidifying paraffin. In the third step, a microtome is used to cut the blocks approximately 300 μm from the root cap by placing the bundle in the block using the eosin-labeled roots, and the cuts are placed on the blades for LM. Finally, the sections are dewaxed, and the tissues are laser cut at 40x or 63x magnification. The tubes are stored at − 80 °C until extraction. The entire process, from sample collection to RNA determination, takes only 3 days to obtain high-quality tissue-specific RNAs for downstream RNAseq or RT-ddPCR experiments.

We started with the Takahashi protocol published in 2010. In our first experiments, we rapidly noted that the samples were histologically degraded, and that the RIN was below 3 (see Supplemental Figure S2 depicting the RIN evolution from Takahashi’s protocol to the final improved protocol); in particular, the structure of the root cuts was not preserved. We therefore first sought to identify a protocol that preserves the structure of the root tissue.

### The use of biopsy foam for sample immobilization preserves root tissues

**Fig. 1:**
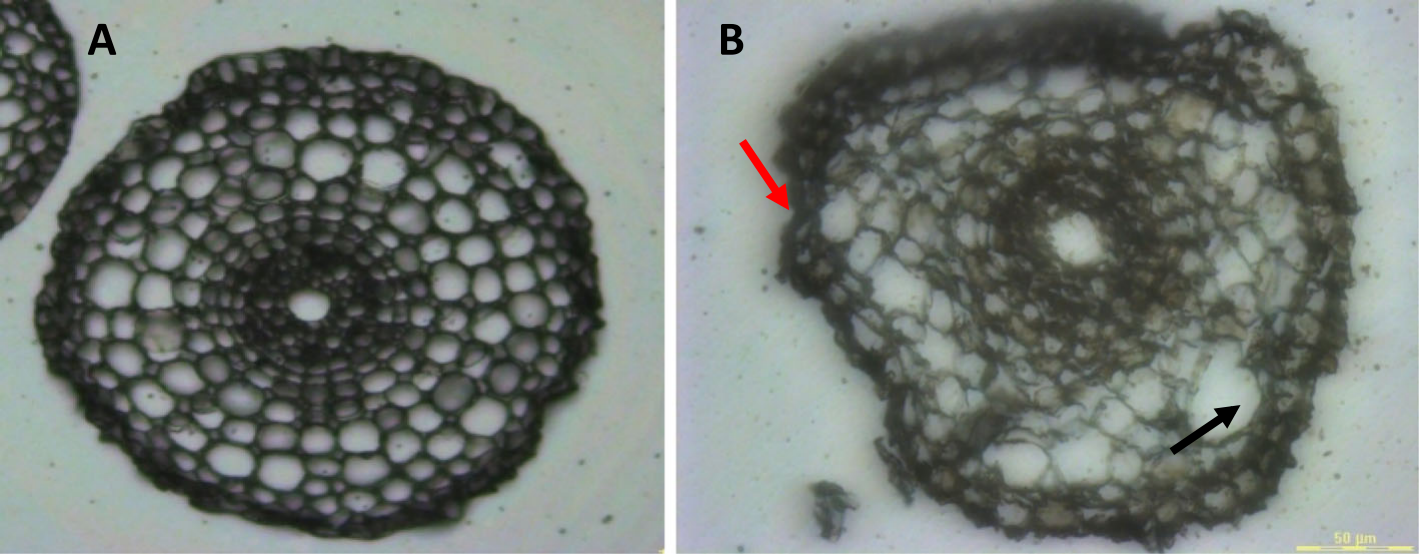
Impact of the use of biopsy foam in the dehydration and embedding steps on root tissue integrity. **a** Root radial section obtained after the dehydration and embedding treatments using biopsy foam. **b** Same as (**a**) without the use of biopsy foam. The root is deformed and no longer circular, and the images show destruction of the most outer cellular layers, which were no longer distinguishable (red arrow), and bursting of more inner cells, such as in the cortex (black arrow). Bar = 50 μm

**Fig. 2:**
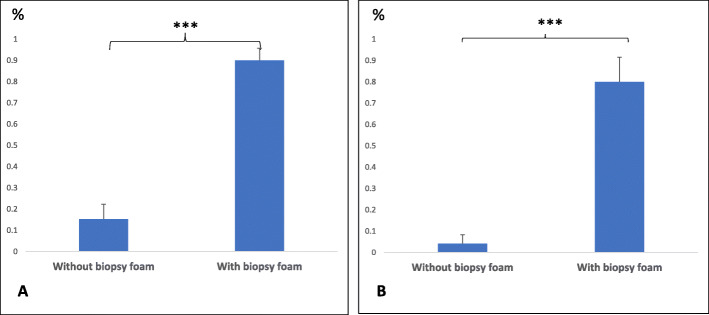
Percentages of crushed and deformed tissues in root tissues with and without biopsy foam. **a** The percentage of crushed external tissue cross sections per total root sections without (left) or with biopsy foam (right). **b** The percentage of round-shaped cross sections per total root sections without (left) or with biopsy foam (right). Bilateral student t-test (***, *p* < 0.001)

We immobilized the root tips collected using biopsy foam when positioning the samples in the embedding cassettes (see Supplemental Figure S1C). Comparison of the sections obtained without foam (Fig. 1a) and those obtained with foam (Fig. 1b) showed that the use of biopsy foam likely prevents movement of the samples during the dehydration and embedding steps as the foam contacts the edges of the cassette or settles between samples to preserve the external tissues and the integrity of the internal tissues (Fig. 2).

### Reducing the duration of the paraffin embedding steps also preserves the integrity of root tissues

**Fig. 3:**
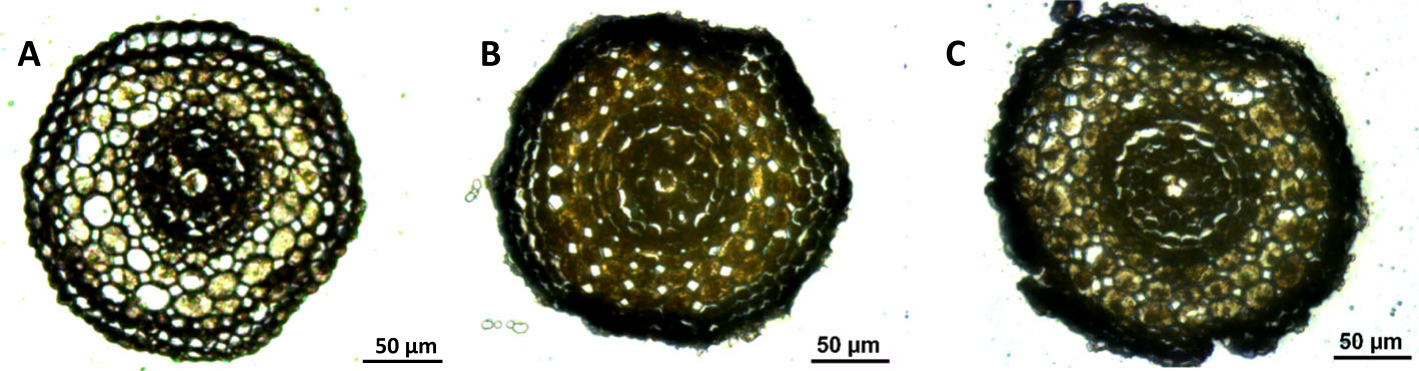
Impact of the embedding time on root tissue integrity. Root cross section obtained after 10 min **a**, 20 min **b** and 30 min **c** of paraffin embedding. Root structure deformation became visible after 20 min **b**, and 30 min **c** resulted in substantial deformation, as demonstrated by a loss of the structure of the external tissues and an inability to distinguish the different external tissues (epidermis, exodermis and sclerenchyma). In contrast, an embedding time in paraffin of 10 min (**a**) yielded tissues without any visible deformation

The use of biopsy foam limited root tip degradation, but the external tissues were still damaged (data not shown). We therefore sought to reduce the embedding time and measure its effect based on the assumption that the heat contact time gradually degrades external tissues. Figure 3 shows the effect of the embedding time on external tissues. All the structures were preserved after 10 min of embedding (Fig. 3a), and the external tissues were partially (Fig. 3b) or completely collapsed (Fig. 3c) after 20 and 30 min of embedding, respectively.

### The embedding time affects the quantity but not the quality of extracted RNA

**Fig. 4:**
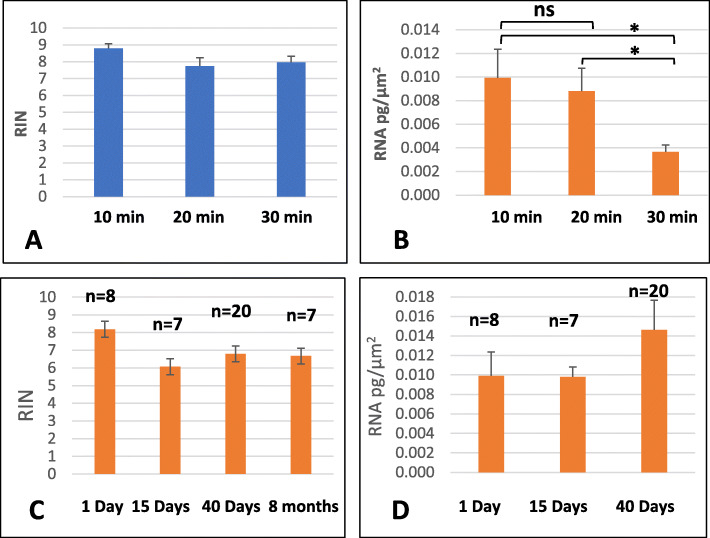
Effects of the embedding time and paraffin block storage on the quality and quantity of extracted RNA from LM root cross sections. Effects of the paraffin embedding time on the quality of extracted RNA **a**) and the amount of extracted RNA (pg/μm^2^) **b**). The data are shown as the means and standard errors calculated from six biological repeats. The effects of the storage time of paraffin blocks at 4 °C on the quality of extracted RNA (RIN) **c**) and the amount of extracted RNA (pg/μm^2^) **d**). Bilateral student t-test (*, *p* < 0.1, ns = not significantly different)

All root tissues were cut with LM after 10, 20 and 35 min of paraffin embedding, and the quality (RIN) and quantity (pg/μm^2^) of the extracted RNA were assessed. First, 10 min of paraffin embedding allowed the extraction of very high-quality RNA (RIN between 8 and 9, Fig. 4a), and high-quality RNA was also obtained with 20 and 30 min of embedding (RIN of approximately 8, not significantly different). Usually, paraffin inclusion time is negatively correlated with the quality of extracted RNA [11]. Our longest inclusion time, 30 min, is short compared to most published protocols (see for instance [9] with 5 h embedding time) but this does not exclude negative correlation with longer inclusion times. Increasing the embedding time decreased the amount of extracted RNA per unit area; specifically, the amount decreased from 0.010 pg/μm^2^ with 10 min of embedding to 0.0038 pg/μm^2^ with 30 min of embedding (*p* < 0.01), resulting in a decrease of more than half (Fig. 4b). We therefore set the duration of the paraffin embedding step to 10 min in the following experiments.

### RNA can be extracted without any loss of quality from paraffin blocks stored for longer than 6 months

Our institute works with many tropical species, and there is often a delay from the time of sample collection in the field to their analysis. In addition, decoupling the paraffin embedding step from the LM cutting step to allow storage of the samples and making LM cuts only when this device, which is generally accessible through shared platforms, is available are desirable. We therefore tested whether the storage of paraffin blocks at 4 °C altered the quality and quantity of the extracted RNA. The qualities of the RNA samples extracted from paraffin blocks after 15 days, 40 days or 8 months of storage at 4 °C were equivalent to those obtained from 1-day blocks (Fig. 4c, RIN values of 8, 6.5, 7 and 7, respectively, not significantly different). The RNA amounts extracted 1, 15 and 40 days after embedding in paraffin were also very similar, with values of 0.01 pg/μm^2^, 0.01 pg/μm^2^ and 0.014 pg/μm^2^, respectively (Fig. 4d).

### The quality and quantity of extracted RNA are correlated with the amount of tissue collected

**Fig. 5:**
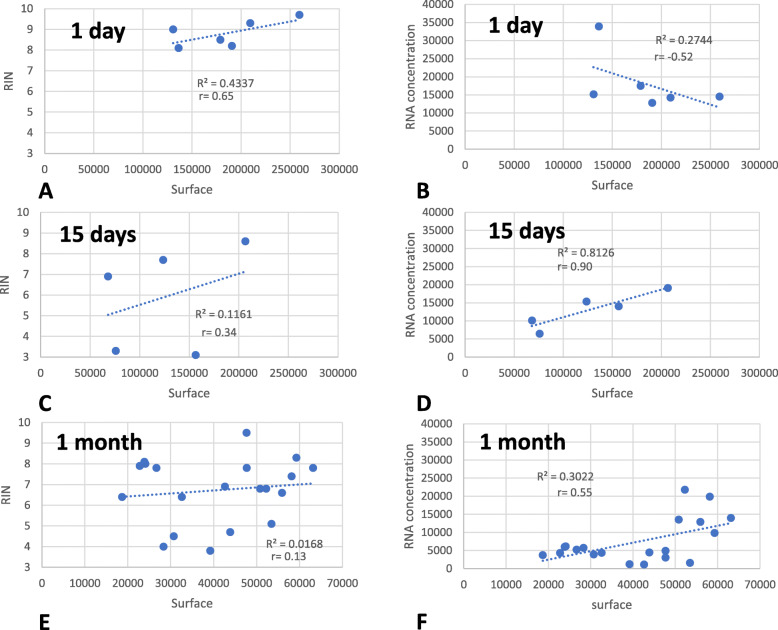
Correlations between the surface of microdissected tissue and the quantity and quality of extracted RNA (*n* = 6). **a**, **c**, **e** The quality of extracted RNA (RIN) as a function of the surface area of microdissected tissue (μm^2^) after 1, 15 and 30 days of paraffin block storage at 4 °**c**. **b**, **d**, **f** The quantity of extracted RNA (pg) as a function of the surface area of microdissected tissue (μm^2^) after 1, 15 and 30 days of paraffin block storage at 4 °C

We attempted to determine whether collecting a greater amount of tissue would increase the quality and quantity of the extracted RNA, which would allow identification of a minimum surface area for future LM experiments. We used RNA extracted from paraffin blocks stored for 1 day, 15 days and 30 days (Fig. 5). First, we observed a slight but non-significant increase in RNA quality with an increasing amount of tissue (Fig. 5a, c and e). Most of the extracted RNA had a RIN higher than 7 despite a few poor-quality extraction products. As expected, we also observed a positive correlation between the RNA quantity and tissue quantity (Fig. 5d, f) except for in the 1-day storage block (Fig. 5b), which was probably due to a stochastic effect of a single outlier (Fig. 5B). Overall, we achieved a RIN greater than 7, reflecting a largely sufficient RNA quality for RNAseq or RT-ddPCR applications.

### Our optimized LM protocol combined with ddPCR offers a complementary tool to in situ hybridization and/or RT-PCR in situ experiments

We used the RT-PCR ddPCR kit from Bio-Rad (See Table 1 for probes and primers) to assay the feasibility of profiling genes from LM-derived samples with a low RNA quantity. This technology is highly sensitive and specific and can be applied to samples with degraded RNA and a very low RNA amount.

**Table 1:** Primers and probes for digital RT-PCR

Tissue	Position	Gene	Primer name	Oligo sequence	Amplicon size	Probe name	Probe sequence	Tm probe	Fluorophore	Reference
All	LOC_Os01g70380	Serine palmitoyltransferase	Serine_F	TTGCCGTCGATAATCCTGAC	196	pSerine	CCTCGTTCGTTCGTCGCTGACGGC	64.2	HEX	Sato et al. 2013 [15]
			Serine_R	GAGGAAGAGGTCGTCAATGG						
	TFIIE (LOC_Os10g25770)	Transcription factor 2E	TFIIE_F	TTAGCTGTGTTGGTCATGGG	161	pTFIIE	CGGAAGAGCTGCTTCAGGTCATCGTCG	63	HEX	This work
			TFIIE_R	TCCCAGGAGGACATTGTGTA						
	EXP’ (LOC_Os07g02340)	Expressed	Exp_F	ATGGGCAGAAGTCGAAGATG	155	pExp	AGCCAGCTTGAGGCCAACAAGAAGGCC	64.9	HEX	This work
			Exp_R	TTTGCACTTGGTCTCAGAGG						
Stele	LOC_Os08g44750	Nodulin-like protein	5NG4_F	GCAGATATGGTGCATCGACA	170	p5NG4	GCCTCCCTCACCCTCGGCGAGAGC	66.4	FAM	Sato et al. 2013 [15]
			5NG4_R	CCCAGAGGACGAGGTAGAG						
	OsSHR1 (LOC_Os07g39820)	SHR1	SHR1_F	CAAGCCGCCTCCG	79	pSHR1	CGTCCTACAACTCGAGG	70	HEX	Henry et al. 2017 [16]
			SHR1_R	TGGACCCGCTCGAC						
Cortex	LOC_Os10g18820	Plant disease response protein	Dis_F	AAGGGATCCACACTTCAGGT	152	pDis	GCTGCAAGCAGTGGTGAGTGGTCTGTT	63.2	FAM	Sato et al. 2013 [15]
			Dis_R	AGTTCTCGAACAGCATCCTC						
	LOC_Os06g48950	OsARF19	OsARR19_F	TCCTCAGACTCAGAACACCA	177	pARF19	TGCCTGGGCTGAGCTTGGTTCAGTGG	64.6	FAM	Yamauchi et al. 2019 [17]; Takehisa et al. 2012 [1]
			OsARR19_R	GGTTCTGCAGGCATAATTGC						
	LOC_Os01g60960	OsLBD1–8	OsLBD1–8_F	CGTCCAAGTCCATATCACCG	198	pLBD1–8	CTTCGCCGCTCCTCCTCCTCCTCC	66.4	FAM	Yamauchi et al. 2019 [17]
			OsLBD1–8_R	TTGAGGGAGCTGTAGTCAGT						
Outer	LOC_Os10g39890	Pollen Ole 1 allergen	Ole_F	TTCTACTTCACCCTGTCCCA	179	pOle	GGACGGTGCCACCTACTGATCGACCGT	65.2	FAM	Sato et al. 2013 [15]
			Ole_R	ACAAAGGCCAAACAACACAC						
	LOC_Os02g06290	OsHAC4	OsHAC4_F	GGAAGGAGAAGAACCCACAC	188	pHAC4	AGGTGTGCGATCCAGGCTCGCGA	64.5	FAM	Xu et al. 2017 [18]
			OsHAC4_R	CTGGCTTTCACTTCGGAGAA						
	LOC_Os06g44970	OsPIN2	OsPIN2_F	CCAGAGCGTCATCTGGTACA	80	pPIN2	CCCTCATGCTCTTCCTCTTCG	63.6	FAM	Wang et al. 2018 [19]
			OsPIN2_R	GGAACTGCTCGGAGATGAG						

**Fig. 6:**
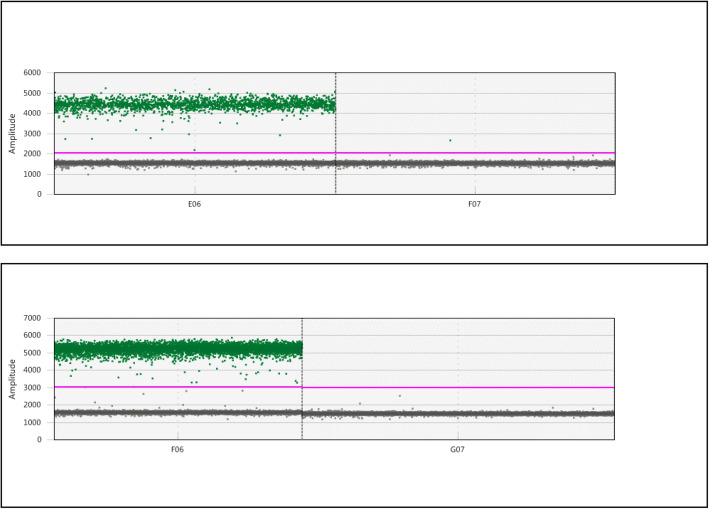
Validation of constitutive control for ddRT-PCR. One-dimensional diagrams of ddRT-PCR for *TFIIE* (up) and *Exp’* (down). The red bar shows the threshold for detection of a positive droplet. One nanogram of root RNA was used as the input on the right image. The left image corresponds to negative control without RNA

Two genes were first tested as constitutive controls for expression analysis by RT-ddPCR (Fig. 6): *TFIIE,* a class IIE transcription factor that is assumed to be constitutively expressed in all transcriptionally active cells, and *EXP’*, a gene with unknown function that was previously identified as a uniformly expressed gene based on a microarray expression dataset [20] (Table 1). Both genes generated only one or two positive droplets in the negative control. From 1 ng of total root RNA, *TFIIE* (Fig. 6a) and *EXP’* (Fig. 6b) generated 2215 and 5814 positive droplets among 13,023 and 13,473 droplets, respectively, which indicates that these genes are expressed at sufficiently high levels to be used as standardization controls for small amounts of RNA, such as those obtained using LM. In addition, *EXP’* is expressed at a higher level compared with *TFIIE* (Fig. 6).

**Fig. 7:**
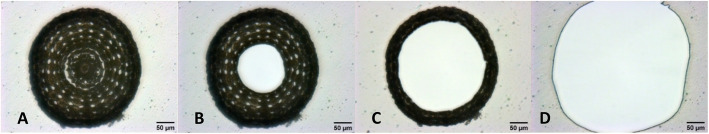
Microdissection of root tissues. **a**-**d** Cutting of the root tissues by microdissection; the tissues are extracted successively from the inside to the outside. **a** A paraffin section before cutting. **b** After cutting the stele + endodermis. **c** After cutting the cortex. **d** After cutting the external tissues, epidermis/exodermis and sclerenchyma

**Fig. 8:**
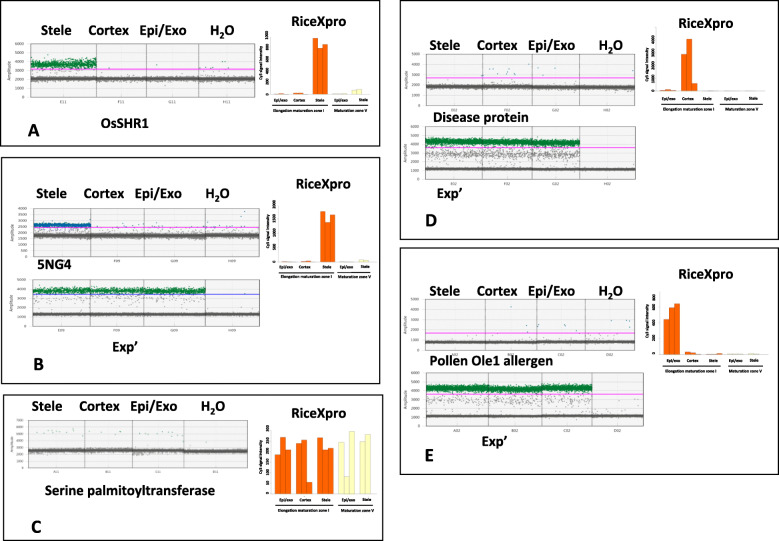
Expression profiling of candidate genes identified from RiceXpro in root tissues using RT-ddPCR. One-dimensional diagrams of ddRT-PCR for *OsSHR1* (**a**)*, 5NG4* (**b**), *serine palmitoyltransferase* (**c**), *disease resistance response protein* (**d**) and *pollen Oe1* (**e**) in three tissues (stele, cortex, and epidermis/exodermis) and a negative control (H_2_O). The expression profiles extracted from the RiceXpro database are shown in parallel. *OsSHR1* was used as a positive control of stele-specific expression. *EXP’* was used as a constitutive control for ddRT-PCR, excepted for OsSHR1 and serine palmitoyltransferase as the probes for these two genes had the same FAM fluorophore as *EXP*’

We successively microdissected three root tissues, stele+endodermis, cortex and outer cell layers (epidermis+exodermis+sclerenchyma) on approximately 30 roots using our LM protocol (Fig. 7). To validate our tissue-specific data, we used *OsSHR1* as a specific tissue control (Fig. 8a). In situ hybridization experiments [21] have revealed that *OsSHR1* is expressed specifically in the stele, and our results confirm that *OsSHR1* is almost exclusively expressed in the stele and expressed at much lower levels, albeit still easily detectable by ddRT-PCR, in the cortex and outer cell layers (Fig. 8a), confirming the absence or a low level of tissue inter-contamination.

### Application of LM and ddRT-PCR for validation of tissue-specific candidate genes

Our aim was to screen candidate expression profiles to identify tissue-specific genes using bibliography sources.

We first selected four genes with potentially distinguishable tissue-specific profiles based on data detailed in the RiceXpro database [14, 15] (Supplemental Figure S3) in addition to *OsSHR1*, which is also predicted to be a stele-specific gene in RiceXpro: i) *5NG4*, specifically expressed in the stele; ii) *serine palmitoyltransferase* (*SP*), expressed in all tissues; iii) *pollen Ole1* (*PO*), expressed in outer tissues (epidermis, exodermis, and sclerenchyma); and iv) *disease resistance response protein* (*DP*), expressed in the cortex. We confirmed the strong stele-specific expression of the *5NG4* gene (Fig. 8b), whereas *SP*, *DP* and *PO* were weakly expressed in this tissue (Fig. 8c, d and e). As expected, PO appeared to be expressed in the epidermis/exodermis (Fig. 8e), SP was expressed at the same low level in all the tissues (Fig. 8c), and DP appeared to be weakly expressed in cortex and outer cell layers (epidermis/exodermis/sclerenchyma) (Fig. 8d). Very few positive droplets in water are often visible for some probes (i.e., 5NG4 and OsSHR1), while droplets are missing in water control for others such as EXP’ even though the gene is highly expressed, suggesting that these few positive droplets more likely result from autohydrolysis of the Taqman probe than from RNA or cDNA contamination of water. The four genes were initially selected due to their similar expression levels, which equaled approximately 1000–2000 as estimated by microarray signals and detailed in the RiceXpro database [14, 15] (Fig. 8 and Supplemental Figure S3), but a poor correlation was found between the levels included in the RiceXpro database and the real expression levels [14, 15].

**Fig. 9:**
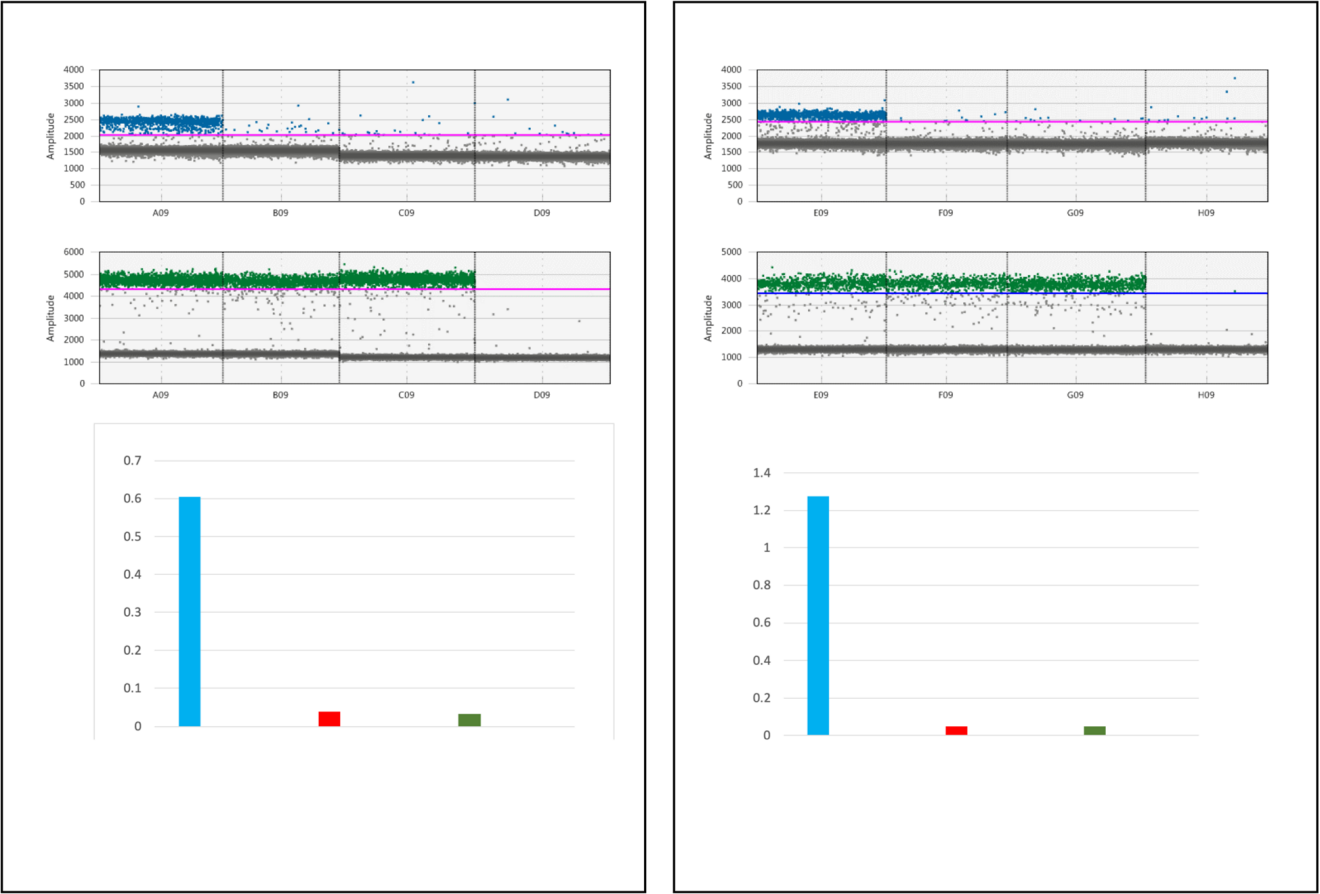
Relative expression profiling of *5NG4* in the stele, cortex and epidermis/exodermis using *TFIIE* and *EXP’* for normalization of RNA quantity. 1D diagram of ddRT-PCR for *5NG4* expression in three tissues (stele, cortex and epidermis/exodermis) and a negative control (water) using *EXP’* (**a**) and *TFIIe* (**b**) for normalization. Blue, FAM probes; green, HEX probes. Bottom, relative expression levels in the three tissues after normalization with *EXP’* (left) or *TFIIE* (right). The abscises represent arbitrary expression values

We performed two RT-ddPCR experiments to estimate the relative expression levels of the *5NG4* gene (FAM probe) among the stele, cortex and external tissues using the *TFIIe* and *EXP’* genes (HEX probe) (Fig. 9) as normalization controls. *5NG4* is preferentially expressed in the stele but is also expressed at a detectable level in other tissues. Normalization using *EXP’* showed that the *5NG4* expression level in the cortex and outer tissues was 15- and 18-fold lower than that in the stele. In contrast, normalization using *TFIIe* revealed that the *5NG4* expression level in the cortex and external tissues was 26- and 25-fold lower than that in the stele.

**Fig. 10:**
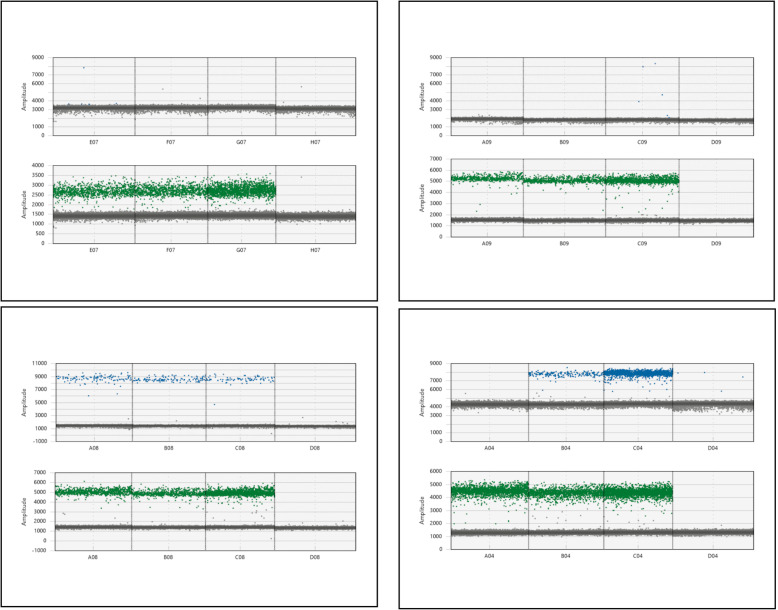
Expression profiling of candidate genes identified from bibliography sources. One-dimensional diagrams of ddRT-PCR for *OsLBD1–8* (**a**)*, OsARF19* (**b**), *OsHAC4* (**c**), and *OsPIN2* (**d**) in three tissues (stele, cortex, and epidermis/exodermis) and a negative control (H_2_O). *EXP’* was used as a constitutive control for ddRT-PCR

To identify new tissue-specific markers, we searched the bibliography sources for candidate genes with expression profiles specific to either external tissues or the cortex and expression in the root tip. In a recent article, the *OsARF19* and *OsLBD1–8* genes have been described as mainly and strongly expressed in the root cortex [17]. Moreover, these genes appear to play an important role in the formation of root aerenchymas through auxin action. The authors also used LM to separate the cortex from the stele but did not isolate the outer tissues. We wanted to confirm the cortex-specific majority expression profiles of *LBD1–8* and *ARF19* and to verify whether they were expressed in external tissues. We did not detect the expression of *LBD1–8* in our conditions (Fig. 10a). In [17], the *LBD1–8* gene is described as mainly expressed between 0 and 5 mm from the root tip and mainly in the cortex beyond 18 mm. We did not find detectable expression of *LBD1–8* (Fig. 10a), which can be explained by the different growing conditions and by the different area sampled for LM in our conditions compared to [17]. *ARF19* expression was detected under our conditions with a similar expression profile between cortex, stele and external tissues (Fig. 10b). *ARF19* does not appear to be mainly and specifically expressed in the cortex, at least not in the first 15 mm of the root tip, under our conditions (Fig. 10b). Therefore, we can conclude that *LBD1–8* and *ARF19* are not cortex-specific markers in our conditions.

We also analyzed the expression of two genes with potentially specific expression profiles in external tissues and root tips, *OsHAC4* and *OsPIN2*. *OsHAC4* plays a role in tolerance to arsenic in rice [18] and appears to be strongly expressed in the expidermis and exodermis, at least in experiments using GUS promoter fusions. Under our conditions, *OsHAC4* was expressed specifically but at low levels in outer tissues (Fig. 10c). We also analyzed the expression of *OsPIN2*, which has been described to be mainly and highly expressed in external tissues with some expression in the cortex [19]. We confirmed these results. *OsPIN2* is strongly expressed in external tissues and at a lower level in the cortex (Fig. 10d) but is absent from stele tissues.

In conclusion, we confirmed only stele-specific expression of *5NG4* and *SHR1*. The other tested genes have either a very low level of expression (*OsHAC4*) or were expressed in at least two tissues with similar expression levels. Other genes from RiceXpro may need to be screened, or RNAseq libraries may need to be built to identify and test potential new tissue-specific markers. Nevertheless, our combination of ddRT-PCR and LM facilitated easy and rapid quantitative expression profiling for ten genes in rice tissues.

### Sensitivity of ddRT-PCR and RT-qPCR

**Fig. 11:**
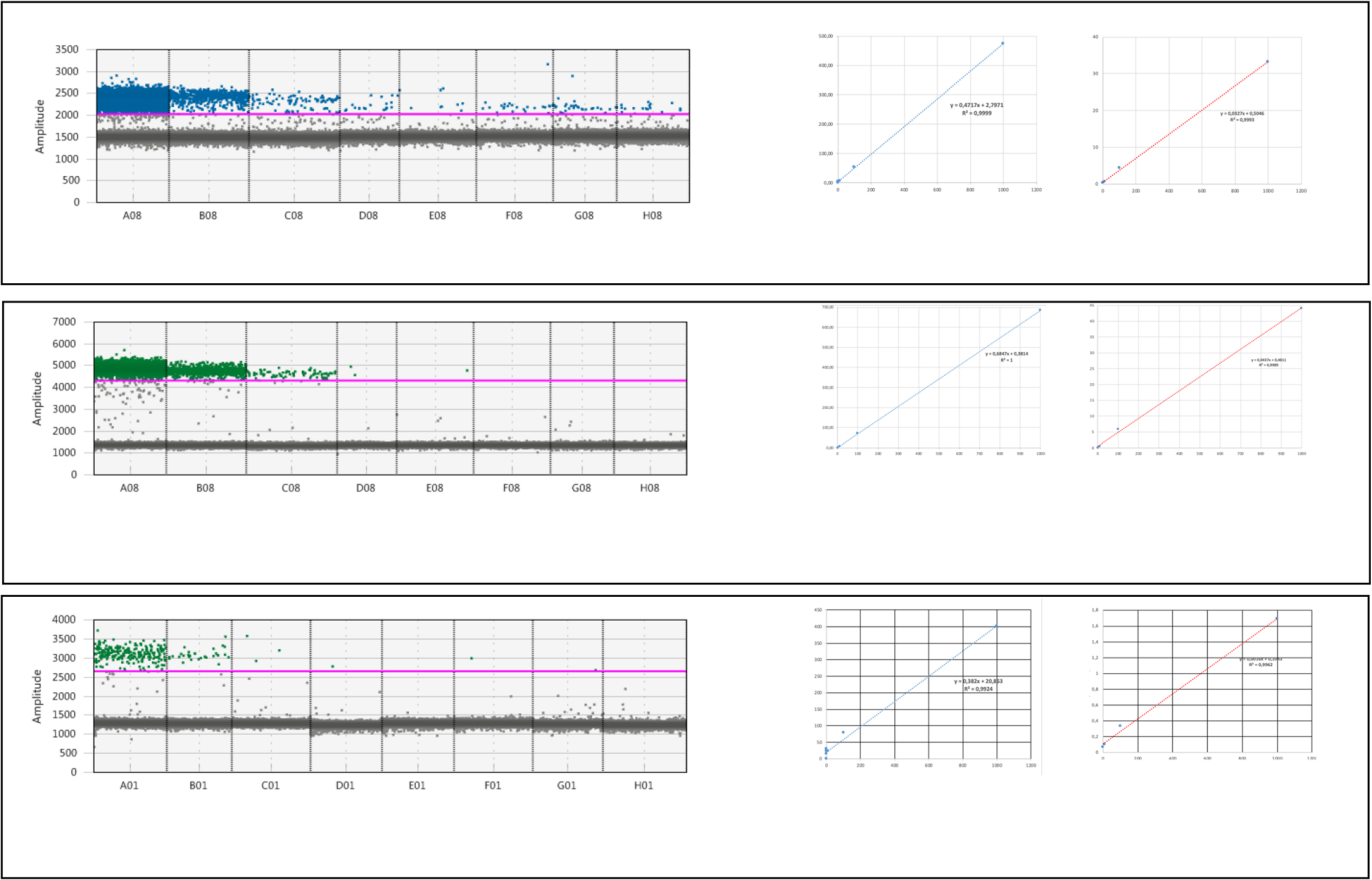
ddRT-PCR for *5NG4* A), *EXP’* B) and *OsSHR1* C) using serial dilutions of total root RNA. Left, 1D diagram of ddRT-PCR for *5NG4*, *EXP’* and *OsSHR1* in serial dilutions of total root RNA ranging from 1 ng to 1 fg and in a negative control (H_2_O). Right, diagrams showing the correlations of the percentages of positive droplets or copies per microliter with the amount (pg) of RNA per reaction. Red bar, threshold for droplet detection

To test the sensitivity of the RT-ddPCR method, we performed serial dilutions of total root RNA to obtain RNA amounts ranging from 1 ng to 1 fg (Fig. 11). We detected the expression of the *EXP’* gene from RNA samples containing at least 100 fg (only one positive droplet was observed with the sample containing 100 fg of RNA). A perfect linear relationship was detected between the number of positive droplets and the amount of RNA or copy number per microliter up to an RNA amount of 10 pg (Fig. 11). *5NG4* gene expression was also detected from samples containing at least 10 pg of RNA, and a perfect linear relationship was found between the number of positive droplets and the amount of RNA or the number of copies per microliter. In contrast, 100 pg of RNA was necessary for the detection of *OsSHR1* expression.

We also tested the sensitivity of qRT-PCR for the EXP’ and SHR1 genes using a range of dilutions using the same TaqMan probes and the same primer pairs for comparison with ddRT-PCR (Supplemental Figure S5). For SHR1, a linear relationship between Ct values and the amount of RNA in pg was found between 250 ng and 100 pg for qRT-PCR. For EXP’, a linear relationship between Ct values and the amount of RNA in pg was found up to 10 pg (Supplemental Figure S5).

Although the sensitivity of ddRT-PCR appears to be better by a factor of approximately 10, caution should be exercised as the sensitivity thresholds between these methods remain close. Therefore, qRT-PCR using TaqMan probes is a possible alternative for relative quantification of expression profiles between tissues with lower sensitivity. In addition to better sensitivity, calibration curves are not required for ddRT-PCR, allowing absolute quantification of the number of RNA molecules for a given gene.

Using the proposed protocol, root tissue RNA from three biological repeats and 30 rice roots can be collected within 1 week by one individual. Moreover, expression profiling can be completed in one week using ddRT-PCR for at least 10 genes, demonstrating that the combination of RT-ddPCR and LM is complementary to in situ RT-PCR and in situ hybridization for tissue expression profiling.

## Discussion

### A simple and rapid protocol for preparing paraffin blocks and performing LM of root tissues

Using our protocol, it is possible to obtain high-quality RNA from rice root tissue that is suitable for downstream applications, such as RT-ddPCR or RNAseq, within 3 days. Compared with the reference protocols [9], we used thicker and fewer tissue sections with high RNA quality suitable for RT-ddPCR and RNAseq.

One of the critical issues for LM is preservation of the tissue structure. Cryosectioning is often preferred to paraffin-embedded specimens because the activity of RNases is reduced despite preservation of the tissue structure (see for instance [22]). In addition, the use of foam has reduced degradation during the inclusion steps, probably due to mechanical shocks (see Figs. 1 and 2). Furthermore, reducing paraffin impregnation times from 30 min to 10 min also facilitated preservation of the structure of the root tissues (see Figs. 3 and 4) compared to a reference protocol [9]. Moreover, we provide a full detailed protocol with the key points that should be optimized by other research groups to adapt the protocol to other tissue/species. In addition to these specific points, cold dehydration and the use of methanol instead of water for mounting the slides are also important parameters to consider (see also Supplemental Figure S2 for more details).

Moreover, paraffin usually preserves the tissue structure better than freezing medium, and paraffin blocks can also be used for other complementary applications, such as* in situ* hybridization and immunochemistry, to compare, for instance, mRNA and protein localization if required [16]. For example, archiving of paraffin blocks has been used for tumor samples [23] in biomedical research, which is a critical point for crop species such as rice because this feature enables sample collection in the field and storage before analysis in a distant laboratory for agronomical or plant pathology analyses.

Our proposed protocol is a high-throughput approach, and as a result, the protocol allows sample collection and RNA extraction from 100 root sections within 1 or 2 weeks for downstream applications such as RNAseq and ddRT-PCR. The proposed protocol can be used for a broad list of plant species with minimal modifications and/or optimization.

### A simple and efficient protocol that is complementary to in situ hybridization and/or in situ RT-PCR

One of the key experiments for characterizing gene or gene network function involves clarification of the tissue expression of candidate genes. This analysis is usually achieved through promoter fusion and/or in situ hybridization and in situ RT-PCR [24]. The former approach can only be applied for species for which genetic transformation approaches have been developed, and the latter is laborious and probe dependent. In addition, *in situ* RT-PCR is a notably less popular approach [24]. Tissue-specific RNA extraction offers an attractive alternative but was not considered until now as a true alternative because its reproductivity and technicity hampered its widespread use, particularly in combination with qPCR. Here, we provide a substantially simpler and reproductible protocol that should help any research laboratory aiming to perform tissue-specific expression profiling of plant tissues by ddRT-PCR as well as other downstream applications, such as RNAseq.

For this purpose, we validated three of four candidate genes detailed in the RiceXpro database [14, 15]. Moreover, we quantitatively analyzed the expression of *OsSHR1* and demonstrated that this gene is also expressed outside stele tissue, albeit at a much lower level. The expression levels observed by RT-ddPCR (Fig. 8 and Supplemental Figure 3) differ significantly from the levels estimated by microarray [14, 15], and the use of RT-ddPCR provides an absolute and more realistic estimate of the tissue-specific transcription level. We also tested the tissue specificity of *OsARF19* and *LBD1–8*, which are described as being specifically expressed in the cortex [17], and *OsPIN2* and *OsHAC4*, which are specifically expressed in outer cell layers based on [18, 19]. We confirmed the specificity of expression in outer cells for *OsHAC4* despite a very low expression level (Fig. 10c) and the strong expression of OsPIN2 in outer tissues. *OsPIN2* expression was also detected in the cortex but at a lower level than in outer layers. We observed very similar expression profiling in our results (Fig. 10d). Altogether, with only 30 microdissected root sections, we were able to complete expression profiling of twelve genes and estimate their relative expression levels in three root tissues.

This protocol will clearly help democratize the technologies for plant applications and should help researchers better understand tissue- and cell-specific responses during plant development or in response to changing environmental conditions, including pathogen/biostimulant interactions. Our future objective is to build on this work and perform a transcriptomic analysis of the formation of root tissues in rice and identify the gene network involved in aerenchyma formation.

## Conclusions

The protocol developed in this study and the detailed troubleshooting guide provided should allow research laboratories to develop and democratize LM-based tissue-specific approaches combined with RT-ddPCR for the analysis of plants. Thus, the proposed protocol will offer an alternative method for the identification and characterization of cell- and tissue-specific responses. Because the starting materials are embedded in paraffin, the samples can be stored for a long time for additional experiments to confirm the results or provide more precise insights using complementary technologies, such as* in situ* approaches, if needed. Using rice root tissues as an example, we showed that this protocol coupling LM and RT-ddPCR can be used to characterize the tissue-specific responses of the transcription factor *OsSHR1* and to perform tissue-specific expression profiling of twelve candidate genes within less than 2 weeks.

## Methods

### Plant material and growth conditions

Nipponbare seeds were initially ordered from the National Bioresource center (https://shigen.nig.ac.jp/rice/oryzabase/about/nbrpRice) and then multiplied in greenhouses in Montpellier. Two hundred dehusked seeds of *Oryza sativa* cv Nipponbare were surface-sterilized in 50 mL of 70% ethanol for 2 min, rinsed with 50 mL of sterile Milli-Q water and disinfected by dipping in a 50-mL 40% bleach solution (9.6° Cl) diluted with distilled water containing 0.4% Tween 80 (Sigma-Aldrich P4780–500 mL) for 30 min under gentle agitation at room temperature. The seeds were then rinsed seven times with 40 mL of distilled water. Fifty seeds were added per petri dish (90 × 14 mm) containing Whatman paper prewetted with 8 mL of Milli-Q. Petri dishes were sealed with parafilm and incubated overnight at 28 °C in a growth chamber (12-h light/12-h dark cycle). Four 6-L buckets and floating sieves were disinfected overnight with 12% H_2_O_2_ at room temperature. The entire system was rinsed generously with sterile water. The buckets were filled with osmosis water, and the floating sieves were placed in the buckets. Twenty seeds/sector (four sectors) were added at 3 pm after 30 h of incubation on petri dishes. The seedlings were grown hydroponically (ARALAB, FitoClima 600) in osmotic water for 7 days (see Table 2 for the light/hygrometric conditions and also Supplemental Figure S1). The program is detailed below. The light cycle was started at 10 am, allowing 5 h of heating from the time that the lamps were switched on to the time of sample collection (at 3 pm). The detailed program (60% humidity, temperatures of 28 °C during the day and 24 °C at night) is as follows: Segment 0; Segment 1: Increase the brightness to 10% over 1 min; Segment 2: Increase the brightness to 90% over 59 min; Segment 3: Maintain the brightness at 90% for 10 h (if sowing at 3 pm, start the cycle with Segment 3 at 240 min); Segment 4: Decrease the brightness to 10% over 1 h; Segment 5: Decrease the brightness to 0% over 12 h; Segment 6: Return to Segment 1.

**Table 2:** ARALAB conditions for rice seedling growth

Time	Temperature	Humidity	Light intensity
720 min	23 °C	60%	0%
1 min	27 °C	60%	10%
59 min	27 °C	60%	10 to 90%
600 min	27 °C	60%	90%
60 min	27 °C	60%	90 to 10%

### Sample collection and fixation

All commercial reagents and product references are detailed in Table 3. All steps must be performed under RNase-free conditions. An aluminum sheet was placed on the work surface, gloves and containers should be successively washed with RNaseZAP, ethanol, RNaseZAP and ethanol, and the same procedure should be used for the LM microscope, three small Histos beakers with their covers, one rack for the Histos5 cassette, and three magnetic stirrers. A large Histos beaker should be prepared to serve as a water bath. All materials should be placed in an oven at 54 °C. The EAA solution (ethanol:acetic acid fixing solution 3:1) (> 200 mL) should be prepared and maintained under cold conditions. A volume of 120 mL of the following dehydration solutions was prepared in advance: 75, 80, 85 90, 95, and 100% absolute ethanol, ethanol:butanol 1:1 (v/v) and 100% butanol 100%. Then, 150 mL of butanol:paraffin 1:1 (v/v) was added, and the next day, 75 mL of butanol and 75 mL of melted paraffin were added at 56 °C (see below). These solutions were stored overnight at 4 °C, except for butanol:paraffin, which should be stored at 54 °C. The EAA solution was distributed into four 30-mL tubes plus two 40-mL tubes. One milliliter of 2% eosin was added to two of the 30-mL fixing solution tubes and to one of the 40-mL fixing solution tubes. A large Histos beaker serving as a water bath was filled with distilled water and stored at 45 °C. Root tips with a length of 1.5 cm were hand-dissected in 10 mL of cold (4 °C) RNAsecure reagent-treated water (AM7005, Thermo Fischer Scientific, USA) and placed in 40 mL of cold EAA solution. Several root samples were transferred in EAA with 2% eosin solution to serve as visual controls and to allow orientation of the root sections during cutting in the paraffin blocks. After sample harvest, the EAA solution was replaced by fresh solution and vacuum infiltrated for 5 min (0.6 psi). The EAA solution was replaced again with fresh solution, and the samples were incubated overnight at 4 °C.

**Table 3:** Reagents and materials

Reagent	Chemical formula	Source	Identifier
Absolute ethanol	C_2_H_5_OH	Honeywell, USA	603–002–00-5
Acetic acid	C_2_H_4_O_2_	VWR, USA	0714–2.5 L
Butanol	CH_3_-CH_2_-CH_2_-CH_2_OH	Sigma-Aldrich, USA	B7906-500 ml
Leica-Paraplast XTRA		Leica, Germany	39,603,002
Xylene	C6H4(CH3)2	Sigma-Aldrich, USA	214,736
Nuclease-free water	H_2_O	Ambion, USA	M9932
Eosin	C_20_H_8_Br_4_O_5_	RAL Diagnostics, France	**312,710**
RNAsecure reagent		Thermo Fisher Scientific, USA	AM7005
RNaseZAP		Sigma-Aldrich, USA	R2020-250 ml
Biopsy foam pads		Simport, Canada	M476–1
Biopsy cassette		Square Mesh Cassette, Orange	70,072-O
Histology cassette		Thermo Scientific, USA	12,677,796
PEN membrane glass slide		Leica, Germany	11,505,190
0.6-mL microcentrifuge tubes		Molecular Bioproducts, USA	3454
ddPCR 96-well PCR plates		Bio-Rad, USA	12,001,925
Pierceable foil heat Seal		Bio-Rad, USA	1,814,040
DG8 cartridges		Bio-Rad, USA	1,804,008
DG8 gasket for ddPCR		Bio-Rad, USA	1,863,009
Droplet generation oil for probes		Bio-Rad, USA	1,863,005
ddPCR droplet reader oil		Bio-Rad, USA	1,863,004
ddRT-PCR kit from Bio-Rad		Bio-Rad, USA	186–4021

### Tissue dehydration and embedding (see also Supplemental Figure S4)

The next day, the paraffin-embedding station and Histos 5 were switched on and cleaned in advance. A volume of 150 mL of paraffin was added to two of the beakers maintained at 54 °C. The butanol:paraffin solution was heated at 54 °C. The biopsy cassettes were transferred in a glass petri dish filled with cold 75% ethanol. Biopsy foam (M476–1, Simport, Canada) was added on the cassette (1,267,796 Thermo Scientific, USA). The roots were very carefully placed on the first foam such that all the root tips were aligned without any stacking. Three bundles of roots were added per cassette, with each bundle containing seven roots. An eosin-stained root was added per bundle. A second biopsy foam was put on the roots before closing the cassette.

The samples were then subjected to 5-min baths with increasing ethanol concentrations (75, 80, 85, 90, 95 and 100%), one 10-min bath in an ethanol/butanol (1:1) solution and one 10-min bath in absolute butanol. The samples were transferred to a water bath at 54 °C and then to a histology microwave oven (Histos 5 Rapid Tissue Processor, Milestone, Italy). The samples were then subjected to a 5-min bath in butanol/paraffin (1:1) solution at 54 °C and 300 W and then two 5-min baths in paraffin at 54 °C and 250 W. Prior to the embedding step, the root bundles were rapidly removed from the cassettes while the paraffin was still liquid and transferred to a cold RNase-free surface. The bundles were subsequently transferred vertically and placed upside down in a molding tray (E70182, EMS, USA). The paraffin blocks were maintained at 4 °C and protected from light.

### Microtomy and laser microdissection

LM collector tubes and the PEN membrane slide were placed under UV light for 30 min. Transversal sections with a thickness of 10 μm were cut on an RNase-free microtome (RM2255, Leica, Germany). An eosin-stained root indicates the positions of all the root tips. Both the PEN membrane slide and methanol were prewarmed on a hot plate at 52 °C for 1 min while cutting roots. Sections of meristematic and differentiated root tissues were visually identified through analysis of the first 500 μm after the first root cap cells. The paraffin sections were then mounted on a PEN membrane glass slide (11,505,190, Leica, Germany) prewarmed at 52 °C and containing drops of methanol. The sections were air dried until the methanol evaporated, and the slide was dewaxed through two 2.5-min baths in cold xylene. Once the xylene had evaporated, the slide was immediately processed for LM (LMD7000, Leica, USA) using the following laser settings: for 63x magnification, power 22, aperture 1, speed 8, Balance 20, Head Current 80%, Pulse Frequency 228, Offset 210; and for 40x magnification, Power 21, Aperture 1, Speed 10, Balance 25, Head Current 100%, Pulse Frequency 120, Offset 180. The outer cells layer, cortex and stele tissues were collected by gravity in a 0.5-mL tube cap filled with 25 μL of extraction buffer from the PicoPure® RNA isolation kit (Cat no. KIT0204, ThermoFisher Scientific, USA). The presence of microdissected tissues on tubes was assessed using low magnification (20X). Following the instructions provided with the Arcturus PicoPure kit, the specimens were stored at − 80 °C until RNA extraction.

### RNA extraction and dosage

RNA extraction was performed in accordance with the instruction manual provided with the PicoPure® RNA isolation kit, and this step involved DNase treatment on a column (RNase-Free DNase Set, Cat no. 79254, Qiagen, Germany). The RNA integrity was evaluated using an Agilent 2100 Bioanalyzer system (Cat no. DE72902360, Agilent, USA) with the Agilent RNA 6000 Pico kit (5067–1513, Agilent, USA).

### Identification of tissue-specific candidates

We used the RiceXpro [14, 15] database (http://ricexpro.dna.affrc.go.jp) to identify genes with tissue-specific expression (see Supplemental Figure 3 and Supplemental Table 1). We then designed primers and TaqMan probes for one tissue-specific gene and a reference gene that shows constant expression in all root tissues. The primers and TaqMan probes were designed using Primer3 in accordance to the manufacturer’s recommendations (Bio-Rad, USA) and were validated by PCR using genomic DNA in a final volume of 25 μL, which consisted of 2.5 μL of 10x Taq Mix, 1.5 μL of MgCl_2_ (25 mM), 2 μL of dNTP (10 mM), 1 μL of forward and reverse primers (10 μM) and 0.6 μL of Diamond Taq (TAQ-I021, Eurogentec, Belgium). The PCR conditions in the thermocycler (Eppendorf™ 6,331,000,041) were as follows: 95 °C for 10 min, 40 cycles of 94 °C for 30 s, 60 °C for 60 s, and 72 °C for 60 s), and a final elongation step of 72 °C for 1 min. The PCR products were analyzed and validated using a 1% agarose gel (see Table 1 for primers and probes).

### RT-ddPCR and RT-qPCR for quantification of gene expression

RT-ddPCR was performed in a solution containing 2 μL of RNA (0.5 ng/μL). A reaction volume of 20 μL was used for droplet generation using the RT-ddPCR reaction kit (Bio--Rad, USA), and this volume consisted of 5 μL of RT-ddPCR Supermix, 2 μL of reverse transcriptase, 1 μL of 300 mM DTT, 1 μL of the primer/probe pair (1 μL of FAM primer/probe and 1 μL of HEX primer/probe for relative expression experiments), 2 μL of RNA QSP, and 20 μL of RNase-free water. The samples were transferred to eight-channel disposable droplet-generation cartridges, and 70 μL of droplet generation oil was added. Each cartridge was then loaded into the QX200 droplet generator (Bio-Rad, USA). After droplet generation, 40 μL of the samples was immediately transferred to 96-well PCR plates (ddPCR 96-well PCR plates, Bio-Rad, USA) and sealed with the PX1 plate sealer (Bio-Rad, USA). The RT-ddPCR conditions on a Mastercycler Nexus Gradient (6,331,000,017, Eppendorf, USA) were 60 min at 50 °C, 10 min at 95 °C, 40 cycles of 30 s at 95 °C and 1 min at 57 °C and a final denaturation step of 10 min at 98 °C. The QX200 droplet reader (Bio-Rad, USA) was used to analyze the droplet fluorescence. Each droplet was analyzed individually using a two-color detection system (FAM, HEX). PCR- and PCR-negative droplets were counted to obtain the absolute quantities of the target RNA molecules using QuantaSoft 1.6 Pro (Bio-Rad, USA) software. The results are presented as 1D plots.

RT-qPCR was performed in a solution containing 2 μL of RNA in white Multiwell 96-well plates and sealed with adhesive foil (Roche Molecular Systems Inc., United States). A reaction volume of 10 μL was used for qPCR using the iTaq Universal Probes One-Step Kit (Bio-rad, USA Ref 172–5140). This volume consisted of 5 μL of iTaq universal probes reaction mix, 0.25 μL of iScript advanced reverse transcriptase, 1 μL of the primer/probe pair (1 μL of FAM primer/probe and 1 μL of HEX primer/probe for relative expression experiments), 2 μL of RNA and 1.75 μL of RNase-free water. The RT-qPCR conditions on a Roche LightCycler 480 (Scan Mode Standard) were 10 min at 50 °C, 2 min at 95 °C, 40 cycles 10 s at 95 °C and 1 min at 60 °C (annealing, extension and reading).

## Supplementary information

**Additional file 1: Supplemental Figure S1.** Preparation of root tip samples. A) A hydroponic culture system using a floating net. B) Harvesting of 2-cm-long root tips and staining of a root tip with eosin for the positioning of root bundles. C) Embedding cassette (right) of root tips covered with biopsy foam (left). D) Embedding of the whole root bundle in paraffin.

**Additional file 2: Supplemental Figure S2.** RIN values obtained after laser microdissection of rice root sections before a) and after optimization steps (b-f). a) The original protocol of [9] b) The RIN value obtained after replacing the initial fixation step with a 5-min vacuum step, followed by overnight fixation at 4 °C. Cold fixation achieved an RIN value close to three. c) The RIN value after replacing the microwave dehydration steps by additional dehydration steps at a cold temperature (4 °C); the RIN value achieved is approximately 5. d) The RIN value for the complete protocol obtained using a paraffin coating and 3 × 5 minutes in the microwave instead of 3 × 3 hours. e, f) RIN values obtained for two more repetitions of the complete protocol. The red bar shows an RIN value of 7 as the minimum quality threshold selected for RNA extraction after laser microdissection.

**Additional file 3: Supplemental Figure S3.** Expression profiling of the putative tissue-specific genes extracted from RiceXpro.

**Additional file 4: Supplemental Figure S4.** Preparation of RNase-free material prior to sample dehydration and embedding. a) Histological cassettes. b) to d) Elements of the water bath for the microwave: b) lid, c) beaker, and d) fixing system for histology cassettes. e) Glass Petri dish. f) Tongs. g) Stirrers.

**Additional file 5: Supplemental Figure S5.** qRT-PCR for *OsSHR1* A) and *EXP’* B) using serial dilutions of total root RNA. Diagrams showing the correlations between the Ct and pg of RNA per reaction.

**Additional file 6: Supplemental File 1.** Methods.

**Additional file 7: Supplemental Table 1.** Genes with tissue-specific expression extracted from RiceXpro.

## Data Availability

All the datasets, including videos and photographs, are included in the article and are also available from the corresponding author upon reasonable request.

## Abbreviations

ddPCR: Digital Droplet PCR
LM: Laser Microdissection
ROL: Root Oxygen Loss
FACS: Fluorescence-activated Cell Sorting
INTACT: Isolation of Nuclei Tagged in Specific Cell Types
