# Supplementary figures and images for: A fast, efficient and high-throughput procedure involving laser microdissection and RT droplet digital PCR for tissue-specific expression profiling of rice roots
Source: BMC Mol Cell Biol. 2020 Dec 10;21:92. doi: 10.1186/s12860-020-00312-y (PMC7727186; doi:10.1186/s12860-020-00312-y)

## Slide 1
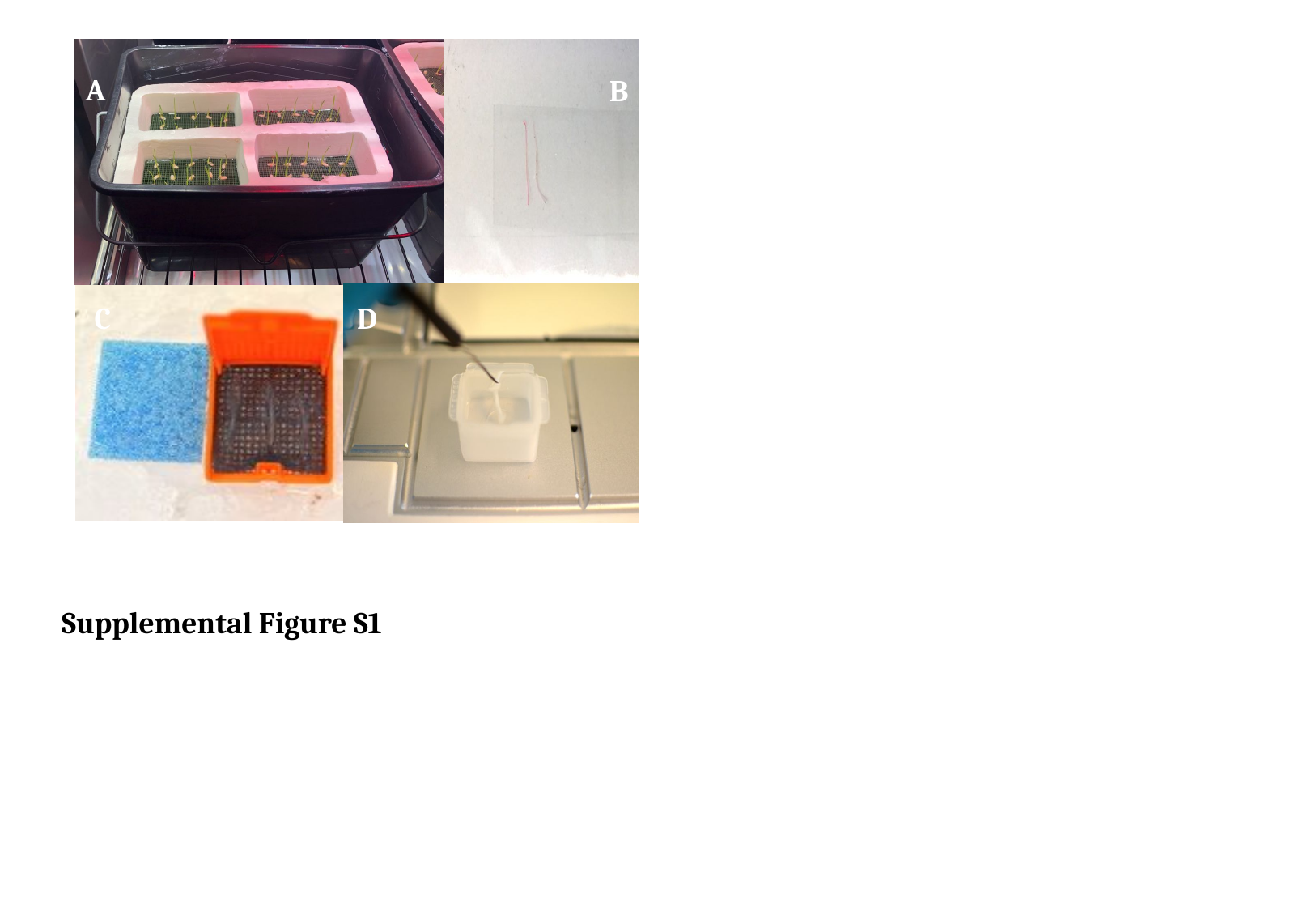

A
B
C
D
Supplemental Figure S1
H
G

Supplement: Supplementary file 1 — Additional file 1: Supplemental Figure S1. Preparation of root tip samples. A) A hydroponic culture system using a floating net. B) Harvesting of 2-cm-long root tips and staining of a root tip with eosin for the positioning of root bundles. C) Embedding cassette (right) of root tips covered with biopsy foam (left). D) Embedding of the whole root bundle in paraffin. [file 12860_2020_312_MOESM1_ESM.pptx]

## Slide 1
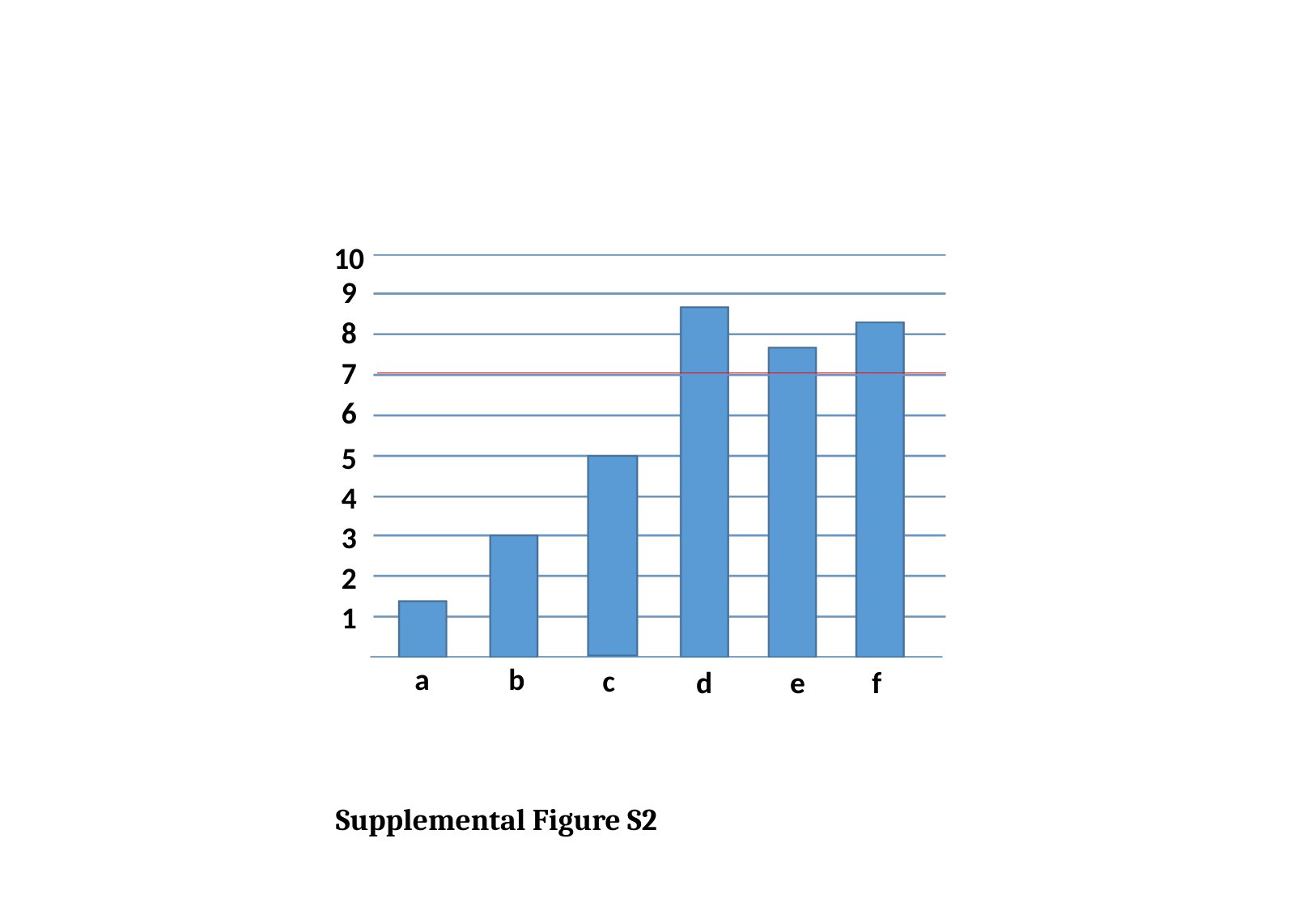

10
9
8
7
6
5
4
3
2
1
a
b
c
d
e
f
Supplemental Figure S2

Supplement: Supplementary file 2 — Additional file 2: Supplemental Figure S2. RIN values obtained after laser microdissection of rice root sections before a) and after optimization steps (b-f). a) The original protocol of [9] b) The RIN value obtained after replacing the initial fixation step with a 5-min vacuum step, followed by overnight fixation at 4 °C. Cold fixation achieved an RIN value close to three. c) The RIN value after replacing the microwave dehydration steps by additional dehydration steps at a cold temperature (4 °C); the RIN value achieved is approximately 5. d) The RIN value for the complete protocol obtained using a paraffin coating and 3 × 5 minutes in the microwave instead of 3 × 3 hours. e, f) RIN values obtained for two more repetitions of the complete protocol. The red bar shows an RIN value of 7 as the minimum quality threshold selected for RNA extraction after laser microdissection. [file 12860_2020_312_MOESM2_ESM.pptx]

## Slide 1
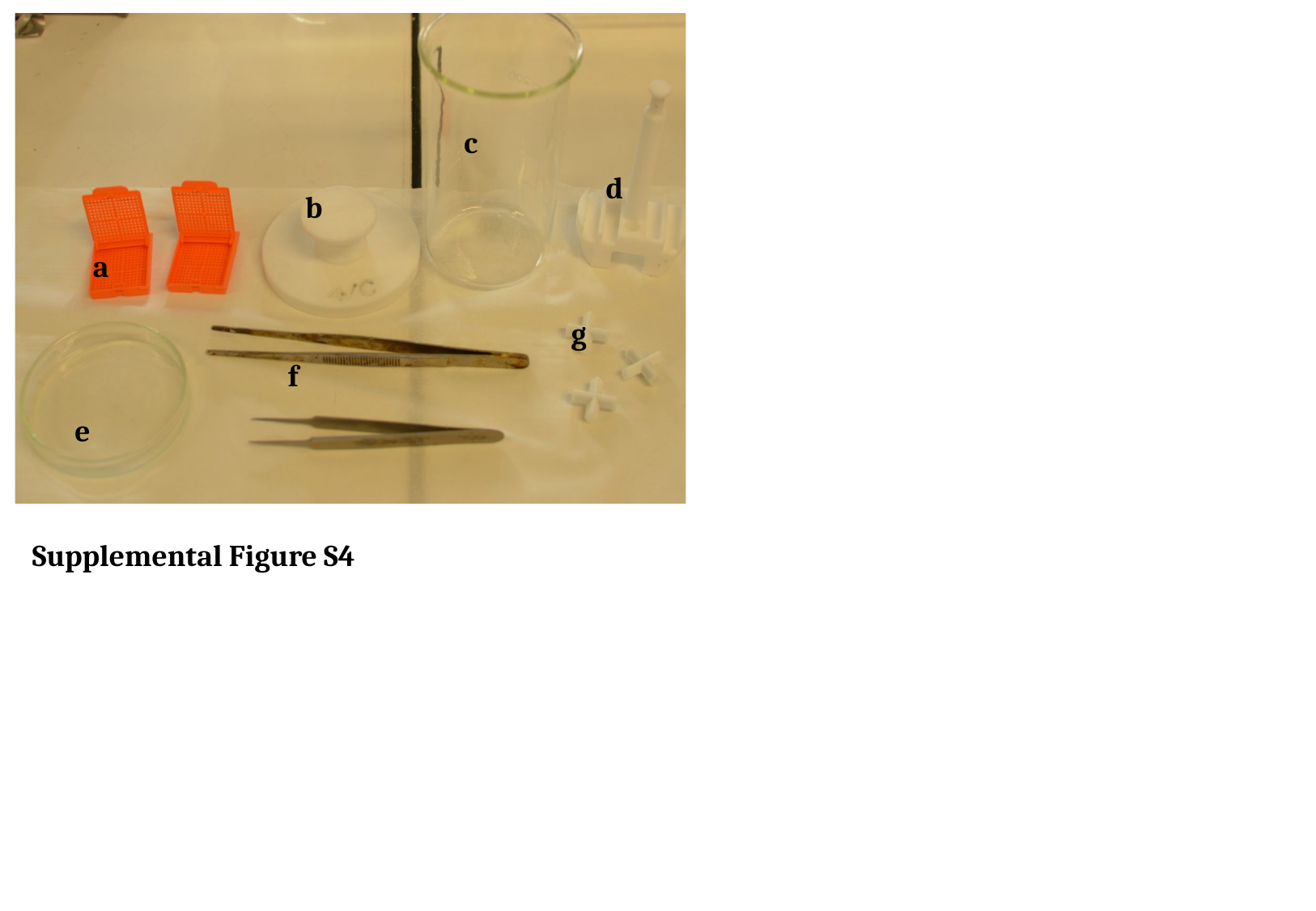

c
d
b
a
g
f
e
Supplemental Figure S4

Supplement: Supplementary file 4 — Additional file 4: Supplemental Figure S4. Preparation of RNase-free material prior to sample dehydration and embedding. a) Histological cassettes. b) to d) Elements of the water bath for the microwave: b) lid, c) beaker, and d) fixing system for histology cassettes. e) Glass Petri dish. f) Tongs. g) Stirrers. [file 12860_2020_312_MOESM4_ESM.pptx]

## Slide 1
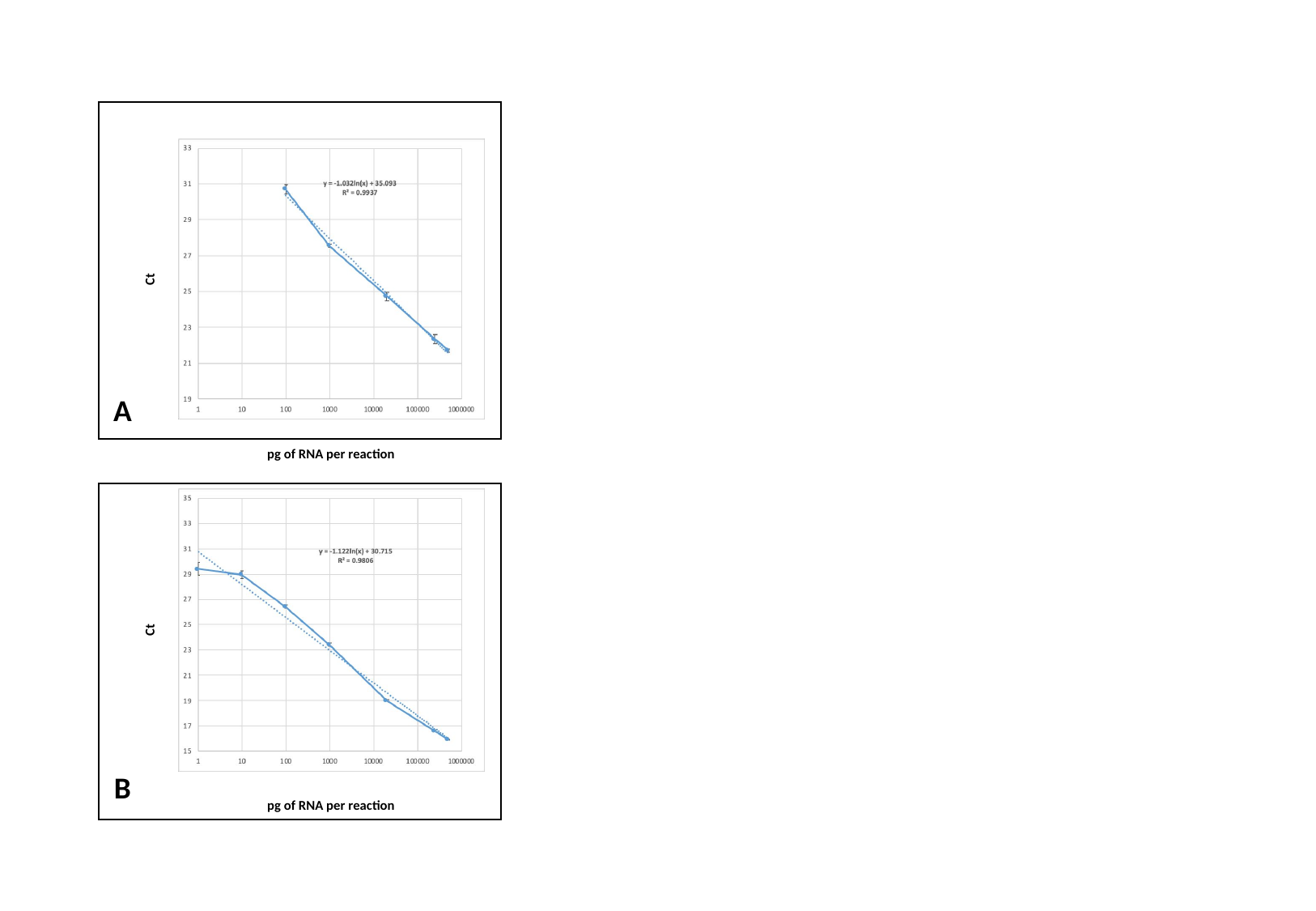

Ct
A
pg of RNA per reaction
Ct
B
pg of RNA per reaction

Supplement: Supplementary file 5 — Additional file 5: Supplemental Figure S5. qRT-PCR for OsSHR1 A) and EXP’ B) using serial dilutions of total root RNA. Diagrams showing the correlations between the Ct and pg of RNA per reaction. [file 12860_2020_312_MOESM5_ESM.pptx]
