# Supplementary material for: A fast, efficient and high-throughput procedure involving laser microdissection and RT droplet digital PCR for tissue-specific expression profiling of rice roots
Source: BMC Mol Cell Biol. 2020 Dec 10;21:92. doi: 10.1186/s12860-020-00312-y (PMC7727186; doi:10.1186/s12860-020-00312-y)
Supplement: Supplementary file 3 — Additional file 3: Supplemental Figure S3. Expression profiling of the putative tissue-specific genes extracted from RiceXpro. [file 12860_2020_312_MOESM3_ESM.pptx]

## Slide 1
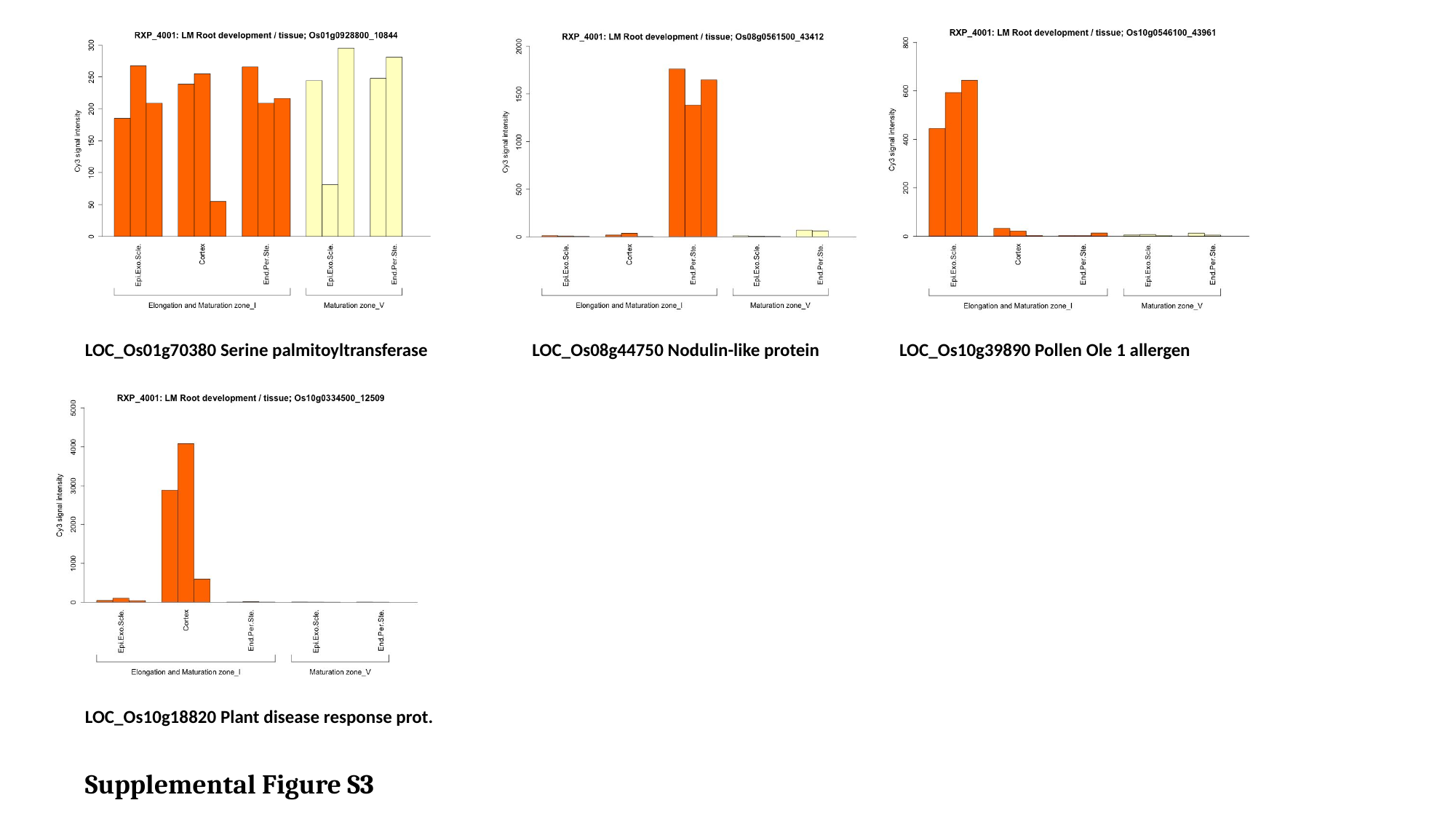

LOC_Os10g39890 Pollen Ole 1 allergen
LOC_Os01g70380 Serine palmitoyltransferase
LOC_Os08g44750 Nodulin-like protein
LOC_Os10g18820 Plant disease response prot.
Supplemental Figure S3
