# Supplementary material for: A fast, efficient and high-throughput procedure involving laser microdissection and RT droplet digital PCR for tissue-specific expression profiling of rice roots
Source: BMC Mol Cell Biol. 2020 Dec 10;21:92. doi: 10.1186/s12860-020-00312-y (PMC7727186; doi:10.1186/s12860-020-00312-y)
Supplement: Supplementary file 6 — Additional file 6: Supplemental File 1. Methods. [file 12860_2020_312_MOESM6_ESM.docx]

**Supplemental File 1: Methods**

We encourage users to let us know by email (perin@cirad.fr) about improvements, clarifications, and troubleshooting that could be added to the protocol. We will regularly update the protocol as we receive feedback from users.

**1. Seedling growth**

*1.1 Seed disinfection* ***(note 1)***

- Select 200 well-formed seeds from 200-300 dehusked rice seeds (**note 1**)

- Disinfect 100-200 seeds in 50 mL of 70% ethanol for 2 min

- Rinse once with 50 mL of Milli-Q water

- Disinfect the seeds in 50 mL of 40% bleach (9.6°) + 2 drops of Tween 20 for 30 min with shaking

- Rinse seven times with 40 mL of Milli-Q water

- Prepare four Petri dishes (90x14 mm) with prewetted Whatman paper and 8 mL of Milli-Q

- Distribute 50 seeds per Petri dish and seal with parafilm

- Incubate O-N at 28°C in a growth chamber (12-h light/12-h dark cycle)

*1.2 Hydroponic system setup and transfer of imbibed seeds (****Supplemental Figure S1*, *note 2)***

*-* Disinfect four 6-L buckets and floating O/N sieves with up to 12% H_2_O_2_ at RT

- Rinse the entire system generously with water, fill the buckets with osmosis water, and place the floating sieves in the buckets

- Fill the sieves with 15-20 grains/sector (four sectors/screen) at 3 pm (after 30 h of incubation)

*1.3 Seedling growth in ARALAB* (see Table 2)

- The program is detailed below. Ideally, the light cycle should be started at 10 am, which would allow 5 h of heating from the time the lamps were switched on to the time of sample collection (at 3 pm).

- The program should be adjusted accordingly.

- The detailed program (60% humidity, temperatures of 28°C during the day and 24°C at night) is the following:

Segment 0

Segment 1: Increase the brightness to 10% over 1 min

Segment 2: Increase the brightness to 90% over 59 min

Segment 3: Maintain the brightness at 90% for 10 h

<-- if sowing at 15 h, start cycle with segment 3 at 240 min -->

Segment 4: Decrease the brightness to 10% over 1 h

Segment 5: Decrease the brightness to 0% over 12 h

Segment 6: Return to segment 1

**2. Root tip harvesting, fixation, dehydration and paraffine embedding (note 3)**

See also Supplemental Figure S4 and Table 3

All steps must be performed under RNase-free conditions. The aluminum sheet placed on the work surface, gloves, and containers should be successively washed with RNaseZAP, ethanol, RNaseZAP and ethanol, and the same procedure should be used for the LM microscope, three small Histos beakers with their covers, one rack for the Histos5 cassette, and three magnetic stirrers. A large Histos beaker should be prepared to serve as a water bath (see "materials" below). All materials should be placed in an oven at 54°C.

*2.1 Solution preparation*

- Prepare the EAA solution (>200 mL) and maintain it under cold conditions

- Distribute the EAA solution into four 30-mL tubes plus two 40-mL tubes

- Add 1 mL of 2% eosin to two of the 30-mL fixing solution tubes and to one of the 40-mL fixing solution tubes

- Prepare 120 mL of the dehydration solutions: 75%, 80%, 85% 90%, 95%, and 100% absolute ethanol, ethanol:butanol 1:1 (v/v) and 100% butanol 100%

- Prepare 150 mL of butanol:paraffin 1:1 (v/v), and the next day, add 75 mL of butanol at 56°C and 75 mL of melted paraffin (see below).

- Store these solutions overnight at 4°C, with the exception of butanol:paraffin, which should be stored at 54°C

- Fill the large Histos beaker that will serve as a water bath and store it at 45°C

*2.2 Root tip harvesting and fixation (under cold conditions)*

- Cut root sections to lengths of at least 1.5 cm and place in an ice-cold Petri dish with 10 mL of RNAsecure reagent-treated water

- Place several roots immediately in the fixing solution with eosin. These roots will serve as landmarks when cutting with the microtome

- Place the remaining roots in another fixing solution without eosin

- Change the fixing solutions with or without eosin, respectively, by pouring carefully.

- Apply a cold vacuum for 5 min

- Replace with 40 mL of the fixing solutions

- Store overnight at 4°C

*2.3 Root tip transfer to cassette*

- Clean and switch on the paraffin embedding station and Histos 5 in advance

- Place 150 mL of paraffin in two of the beakers maintained at 54°C

- Place and maintain the butanol:paraffin solution at 54°C

- Place the biopsy cassettes in a glass Petri dish filled with cold 75% ethanol

- Place a biopsy foam on the cassette

- Very carefully position the roots on the first foam such that all the root tips are aligned without any stacking among them

- Form up to three bundles of roots per cassette (each bundle should contain seven roots)

- Add one eosin-stained root per bundle

- Gently place a second biopsy foam on the roots and close the cassette

*2.4 Sample dehydration*

*Each step should be performed for 5 min on ice*

- 5-min bath in 75% ethanol followed by a 5-min bath in 80% ethanol
- 5-min bath in 85% ethanol following by a 5-min bath in 90% ethanol
- 5-min bath in 80% ethanol followed by a 5-min bath in 85% ethanol
- 5-min bath in 90% ethanol followed by a 5-min bath in 95% ethanol
- 5-min bath in 95% ethanol followed by a 5-min bath in 100% ethanol

*Infiltration: each step should be performed for 10 min on ice*

- Replace the solution of absolute ethanol from the last bath with 50% ethanol/50% n-butanol
- Replace the 50% ethanol/50% n-butanol with 100% n-butanol

*2.4 Paraffine embedding* ***(note 4)***

The microwave cannot be used to directly heat the paraffin. It is thus necessary to directly use a water bath system with the Histos 5 beaker.

- Replace the 100% butanol solution with paraffin:butanol 1:1 (v/v)

- Incubate the sample in the solution for 5 min at 54°C and 300 W

- Transfer the sample to a bath with 100% paraffin

- Incubate the sample in the solution for 5 min at 54°C and 250 W

- Transfer the sample to the second paraffin bath

- Incubate the sample in the solution for 5 min at 56°C and 250 W

- The following steps should be performed in the paraffin embedding station.

- Transfer the samples into a Petri dish with 100% paraffin in the heating compartment

- Gently grasp the root bundles by the ends opposite the tips

- Gently pinch the root tips together while the paraffin remains as a liquid

- Place the root tips on an RNase-free surface

- Remove all root bundles before embedding under RNase-free conditions

- Include the roots in paraffin by keeping them straight and pointed toward the bottom of the prewarmed molding tray

- Store the paraffin block at 4°C protected from the light

**3. Microtome sectioning and LM**

*3.1 Microtome sectioning*

- Treat the LM collector tubes and PEN membrane slide for 30 min under UV light

- Cut 10-µm paraffin sections using the microtome. If the roots are correctly aligned, the eosin-stained root indicates the position of all the root tips

- Start sectioning as close as possible to the root tips and cut up to 1500 µm

- While cutting the sections, warm both the PEN membrane slide and methanol on a hot plate at 52°C during 1 minute

- Mount the sections on the PEN slide by adding a few drops of methanol (**note 6**)

- Wait until the methanol has evaporated completely

*3.2 LM* ***(note 7)***

- Remove the paraffin through two 2-min washes in cold xylene

- Air dry the slide until the xylene has completely evaporated

- Microdissect the stele, endoderm and cortex

- Collect the samples in specially designed tubes containing 25 µL of extraction buffer (**notes 8, 9, and 10**)

- Follow the instructions provided with the Arcturus PicoPure kit

- Store at -80°C until RNA extraction

*3.3 RNA isolation, quantification and quality assay*

- Extract RNA using the Arcturus PicoPure kit (+ DNasein column (RNase-Free DNase Set, Cat no. 79254, Qiagen, Germany)

- Assess the RNA quality and concentration using an Agilent RNA 6000 Pico kit

**Notes and troubleshooting**

**Note 1:** To achieve homogeneous germination, select the best possible seeds and avoid green and unbroken seeds with fungi and/or possible bacterial contamination.

**Note 2**: Keep the seeds separated throughout the growing process to reduce cross-contamination.

**Note 3**: Handle the samples gently to preserve tissue integrity.

**Note 4:** Minimize the paraffin embedding time to reduce tissue degradation and ensure good RNA quality. For new tissues/species, try several fixation, embedding, and embedding times, and select the shortest fixation time that allows good fixation and paraffin embedding.

**Note 5**: Reduce electrostatic deleterious effects. Static electricity might cause removal of the samples dissected by LM of the cap. Try to keep the level of static electricity as low as possible by maintaining the hygrometry level above 60% during LM at room temperature. Treat all LM materials by exposure to UV light for 30 min. UV should prevent the root sections from dropping from the slide. A correct laser setting is also critical for preventing the accumulation of static electricity.

**Note 6:** Use the lowest possible amount of methanol when mounting the slide. We usually used a pipette and less than 200 µL of methanol per slide. A lower amount of methanol shortens the drying time, blocks RNA degradation and prevents root sections from dropping from the slide. You should proceed to the xylene step once the slide is completely dry.

**Note 7**: Adjusting the laser settings is crucial for proper microdissection. Refer to the microscope manual and find the best setup for your samples.

**Note 8**: We never passed the 2-h limit for sample collection in a single collecting tube to preserve the RNA quality. Nevertheless, it is possible to pool several tubes filled with a low volume of extraction buffer to increase the RNA concentration. Adjust the extraction buffer volume based on the amount of tissue to increase RNA yield.

**Note 9**: We never passed the 4-h limit for microdissection per slide to prevent RNA degradation. Instead of increasing the microdissection time per slide, the samples should be pooled.
